# Ventilation-induced acute kidney injury in acute respiratory failure: Do PEEP levels matter?

**DOI:** 10.1186/s13054-025-05343-5

**Published:** 2025-03-21

**Authors:** Martín H. Benites, Fernando Suarez-Sipmann, Eduardo Kattan, Pablo Cruces, Jaime Retamal

**Affiliations:** 1 Unidad de Pacientes Críticos, Clínica Las Condes, Santiago, Chile; 2 Facultad de Medicina, Escuela de Medicina, Universidad Finis Terrae, Santiago, Chile; 3 Doctorado en Ciencias Médicas, Escuela de Medicina, Pontificia Universidad Católica de Chile, Santiago, Chile; 4 Departamento de Medicina Intensiva, Pontificia Universidad Católica de Chile, Santiago, Chile; 5 CIBER de Enfermedades Respiratorias, Instituto de Salud Carlos III, Madrid, Spain; 6 Department of Intensive Care Medicine, La Princesa University Hospital, Madrid, Spain; 7 Department of Surgical Sciences, Uppsala University, Uppsala, Sweden; 8 Facultad de Ciencias de La Vida, Universidad Andres Bello, Santiago, Chile; 9 Unidad de Paciente Crítico Pediátrico, Hospital El Carmen Dr. Luis Valentín Ferrada, Santiago, Chile

**Keywords:** Acute respiratory failure, Acute kidney injury, Positive end-expiratory pressure, Venous congestion

## Abstract

Acute Respiratory Distress Syndrome (ARDS) is a leading cause of morbidity and mortality among critically ill patients, and mechanical ventilation (MV) plays a critical role in its management. One of the key parameters of MV is the level of positive end-expiratory pressure (PEEP), which helps to maintain an adequate lung functional volume. However, the optimal level of PEEP remains controversial. The classical approach in clinical trials for identifying the optimal PEEP has been to compare “high” and “low” levels in a dichotomous manner. High PEEP can improve lung compliance and significantly enhance oxygenation but has been inconclusive in hard clinical outcomes such as mortality and duration of MV. This discrepancy could be related to the fact that inappropriately high or low PEEP levels may adversely affect other organs, such as the heart, brain, and kidneys, which could counteract its potential beneficial effects on the lung. Patients with ARDS often develop acute kidney injury, which is an independent marker of mortality. Three primary mechanisms have been proposed to explain lung-kidney crosstalk during MV: gas exchange abnormalities, such as hypoxemia and hypercapnia; remote biotrauma; and hemodynamic changes, including reduced venous return and cardiac output. As PEEP levels increase, lung volume expands to a variable extent depending on mechanical response. This dynamic underlies two potential mechanisms that could impair venous return, potentially leading to splanchnic and renal congestion. First, increasing PEEP may enhance lung aeration, particularly in highly recruitable lungs, where previously collapsed alveoli reopen, increasing lung volume and pleural pressure, leading to vena cava compression, which can contribute to systemic venous congestion and abdominal organ impairment function. Second, in lungs with low recruitability, PEEP elevation may induce minimal changes in lung volume while increasing airway pressure, resulting in alveolar overdistension, vascular compression, and increased pulmonary vascular resistance. Therefore, we propose that high PEEP settings can contribute to renal congestion, potentially impairing renal function. This review underscores the need for further rigorous research to validate these perspectives and explore strategies for optimizing PEEP settings while minimizing adverse renal effects.

## Background

**Fig. 1:**
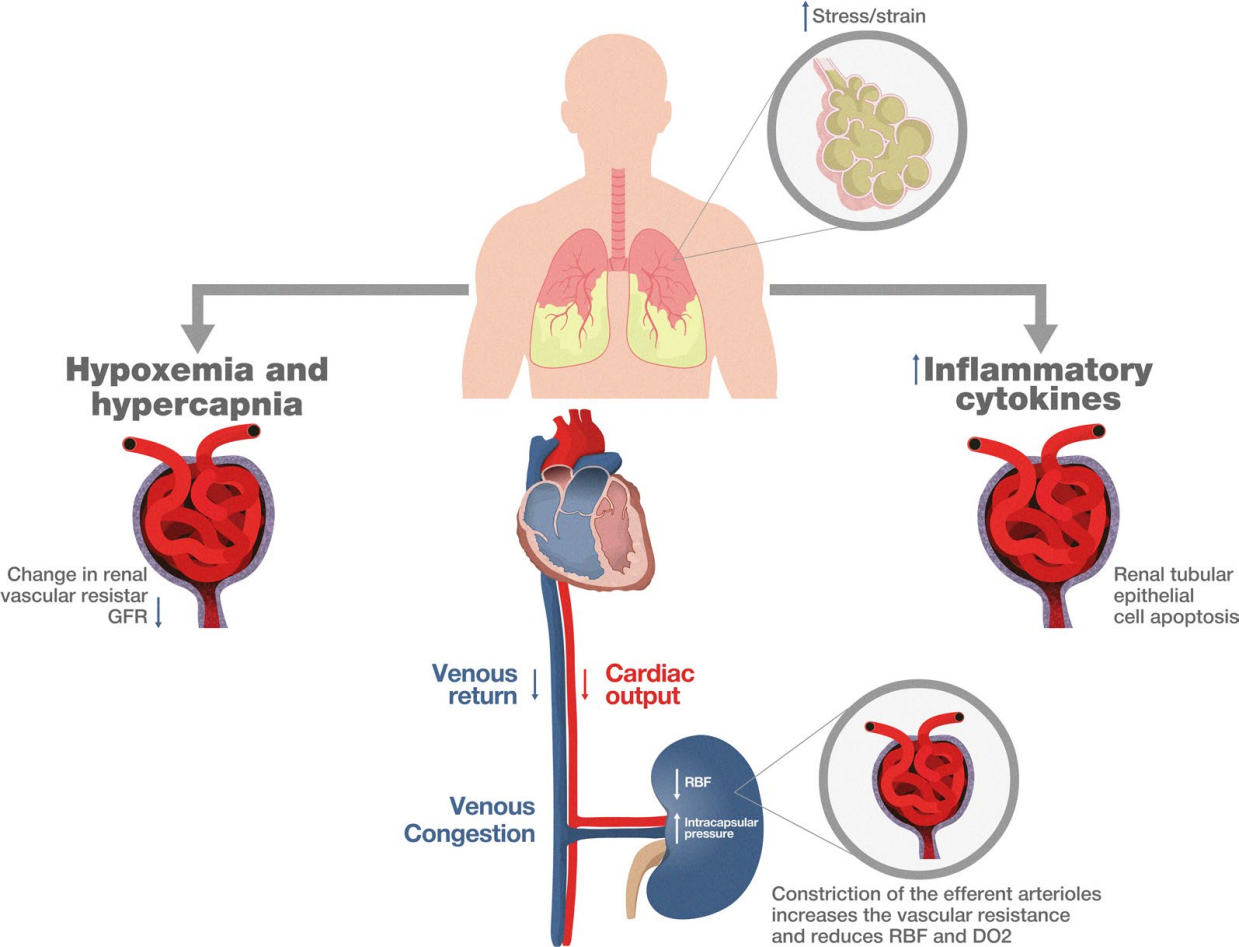
Three mechanisms proposed to explain lung-kidney cross-talk during mechanical ventilation. Abnormalities in gas exchange disrupt renal blood flow. Mechanical ventilation (MV) induces inflammation and damages renal cells. Hemodynamic changes from MV impair venous return and cardiac output, worsening the tubular injury. PEEP-induced venous stasis compromise is linked to acute kidney injury (AKI)

Mechanical ventilation (MV) is associated with the risk of acute kidney injury (AKI) in critically ill patients. Recent studies have shown that patients undergoing MV have an approximately three-fold higher risk of developing AKI than those not requiring ventilatory support [1]. A complete understanding of the mechanisms by which MV influences AKI development represents a clinical challenge. Three main mechanisms have been proposed to explain lung-kidney cross-talk during MV (Fig. 1): first, gas exchange abnormalities, such as hypoxemia and hypercapnia, affect the chemoregulation of the diameter of the afferent renal artery, impairing renal blood flow. Second, MV can trigger the release of inflammatory mediators from the lungs, causing remote damage to renal tubular cells (mechanotransduction & biotrauma) [2]. Finally, hemodynamic changes associated with MV decrease venous return and cardiac output, impairing renal blood flow and causing tubular damage [3]. Among these mechanisms, positive end-expiratory pressure (PEEP)—induced hemodynamic impairment is associated with AKI onset. Experimental and clinical evidence suggests that venous congestion and reduction in renal artery blood flow caused by PEEP are highly plausible mechanistic explanations leading to lung-kidney cross-talk [4]. Other potential pathophysiological factors, such as increased intrarenal pressure, release of hormones that counterregulate renal function, and impaired renal oxygenation and metabolism, could be associated with renal congestion and further contribute to kidney dysfunction during MV [4].

In this review, we address the main evidence and factors supporting the critical role of high PEEP in the development of AKI. In addition, we explain how hydrostatic pressure is transferred from the lungs to the renal venous vascular bed. Given this connection, we deepen our understanding of these mechanisms and suggest future research directions for this topic.

### Effects of PEEP on respiratory system

PEEP is a fundamental component of mechanical ventilation, particularly in managing (ARDS), whose primary role is to induce alveolar recruitment and avoid alveolar collapse [5]. In addition, by increasing aerated lung tissue, PEEP can improve lung compliance, enhance oxygenation, reduce intrapulmonary shunting, and promote redistribution of pulmonary edema fluid from the alveoli to the interstitial space, especially when lung recruitment potential is high [6, 7]. Despite these benefits, careful adjustment of the PEEP is essential to minimize the risk of alveolar overdistension, which is associated with direct structural damage to the lungs, systemic inflammatory responses, and hemodynamic alterations. Optimizing ventilatory parameters to prevent excessive lung deformation is crucial for mitigating these complications while preserving the protective effects of PEEP on the lung parenchyma [7].

These effects underscore PEEP’s crucial role in preventing complications during the treatment of acute respiratory failure. Efforts are ongoing to identify the optimal PEEP level that maximizes physiological benefits and delivers long-term clinical outcomes [8]. Several studies have compared high PEEP strategies with low PEEP strategies using PEEP/FiO2 tables, consistently showing that high PEEP values improve oxygenation and lung compliance [9–11]. However, these benefits did not translate to significant improvements in mortality or ventilator-free days. In addition, the optimal choice of a specific PEEP scale using this table remains controversial because of the lack of a physiological basis, so its clinical use, out of comparative studies, is today questioned.

In turn, in clinical practice, there is no one-size-fits-all rule for adjusting PEEP, and the search continues for the optimal setting that balances its many positive effects on lung function. Recent research suggests that applying high PEEP levels to non-recruitable lung tissue and low levels to recruitable lung tissue significantly increases mortality [12]. Therefore, it is crucial to emphasize that adjustments in PEEP settings can induce lung volume changes of varying magnitudes, depending on the parenchymal response to airway pressure fluctuations [13, 14].

First, a rise in PEEP can significantly enhance lung expansion, an effect amplified under high recruitment conditions, where the applied pressure facilitates the reopening of previously collapsed alveolar units [15]. In this context, pressure transmission to the pleural space [13, 14] may contribute to systemic venous congestion by compressing the vena cava, thereby promoting regional congestion in abdominal organs [16].

Second, changes in lung volume can influence pulmonary vascular resistance, which, in turn, can either facilitate or hinder right ventricular ejection. This relationship has traditionally been depicted as a U-shaped curve, rising from a nadir at functional residual capacity, where both derecruitment and overdistention contribute to an increase in pulmonary vascular resistance [17]. Collapsed lung units exhibit increased vasomotor tone due to hypoxic pulmonary vasoconstriction and promote adverse hemodynamic effects [18]. This response can be reversed by applying appropriate PEEP levels to recruit lung units and subsequently increase the volume of aerated tissue [15].

Conversely, increasing PEEP in lungs with low recruitment ability may induce minimal changes in lung volume while elevating airway pressure [15, 19]. This leads to alveolar overdistension, which can induce alveolar capillary compression and reduce the cross-sectional area of these structures [17]. This, in turn, increases pulmonary vascular resistance, which can be transmitted to the right heart [15, 17, 19]. Therefore, this process may contribute to a right heart failure-like state by elevating the right heart filling pressure and causing retrograde venous congestion, even in the absence of overt right ventricular dysfunction or absolute hypervolemia.

Likewise, overdistension can increase the risk of ventilator-induced lung injury (VILI) and, through systemic inflammatory mediators, damage renal tubules [2]. This indicates that elevated levels of PEEP, even when oxygenation is improved [9–11], could cause unnoticed adverse effects in other organs distant from the lungs [3, 20, 21]. Therefore, the systemic effects of PEEP critically depend on how the lungs react to the changes in pressure (recruitment vs. overdistension vs. collapse) and on the proper selection of an individualized level rather than on the absolute value of the PEEP setting [15, 19]. As a result, kidney function, which accounts for 20% of the total cardiac output [22], is influenced by the systemic effects of PEEP on renal blood flow and congestion. This combination triggers a range of interrelated physiological effects that affect renal performance [23].

### Effects of PEEP on the cardiovascular system

**Fig. 2:**
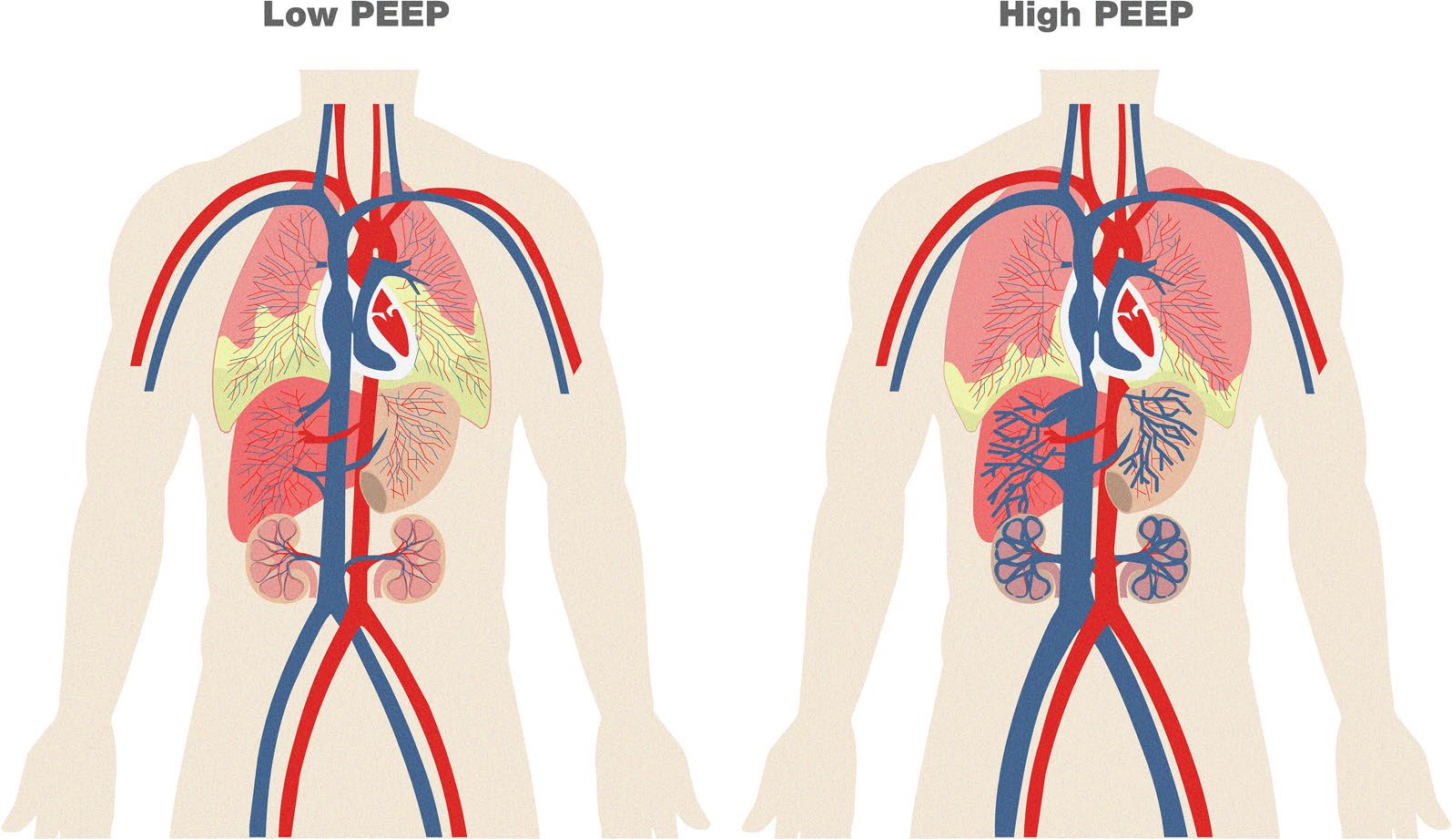
High PEEP levels exacerbate abdominal organ congestion. Proposed systemic effects generated by PEEP levels in patients with acute respiratory failure. Low PEEP levels do not contribute to abdominal venous stasis. High PEEP levels increase the lung volume and may induce extrathoracic venous congestion. Liver, gastrointestinal tract, and kidney congestion are depicted by the prominence of veins (blue color) under high PEEP conditions

PEEP can generate significant hemodynamic effects. These effects can retrogradely impact the systemic venous system and anterogradely affect cardiac output, thereby influencing systemic oxygen transport. An increase in intrathoracic pressure leads to elevated pressure in the right atrium, which results in a decreased pressure gradient for venous return. Consequently, the venous return may be compromised, reducing the right ventricular preload and, thus, compromising systemic organ perfusion [24–27]. Thus, inappropriate PEEP settings applied to lung tissue with low recruitability can directly increase pulmonary vascular resistance, impair right ventricular function, and deteriorate venous return [15], ultimately resulting in systemic venous congestion (Fig. 2). In contrast, in an animal experimental model, and under conditions of high recruitability, an increase in the volume of the right lower lung lobe and diaphragm descent may compress the inferior vena cava [16]. Therefore, in both lungs with low or high reclutability, PEEP could increase venous resistance in large veins as they enter the thorax, interfering with venous return according to Guyton’s equation for venous return: venous return = Mean Systemic Filling Pressure (MSFP)—right atrial pressure (RAP)/vr (venous resistance) [28].

Consequently, both arterial and venous circulation may be compromised in patients with ARDS on MV. The outcome of these physiological changes can thus affect renal perfusion pressure and compromise glomerular filtration rate [3].

Additionally, volemia can exacerbate the PEEP-related hemodynamic effects. Under hypovolemia conditions, increased PEEP amplifies the negative impact on mean arterial pressure (MAP), cardiac output, and renal blood flow [29]. On the other hand, volume overload is also considered a risk factor for AKI [30–32], and it may be further exacerbated in elevated PEEP levels, where increased hydrostatic pressure can intensify congestion in the abdominal organs and impair renal function. These effects underscore the importance of meticulous fluid management and careful monitoring of bedside venous congestion when PEEP is set.

The concept of renal venous congestion has been extensively investigated in the context of heart failure, called cardiorenal syndrome [33], which activates and maintains a vicious cycle of decreased cardiac output, increased filling pressure, and fluid overload. Patients with chronic congestive heart failure also develop concomitant visceral edema that can evolve in severe cases to cirrhosis, terminal renal failure, and severe intestinal dysfunction with increased permeability to endotoxins and bacteria [34].

### Effect of PEEP on splanchnic circulation

PEEP transfers pressure through the diaphragm into the abdominal cavity. In a study by Verzilli et al., changing PEEP from 0 to 12 cmH₂O in a series of ARDS patients resulted in an average increase in intra-abdominal pressure (PIA) by 4 mmHg. This effect was more pronounced in patients with a baseline PIA ≥ 12 mmHg, significantly affecting hemodynamics and respiratory mechanics [35].

Diaphragmatic descent compresses the splanchnic blood reservoir, which consists of blood stored in the intestines, spleen, and liver. This mechanical compression reduces the splanchnic venous capacitance, causing a redistribution of the blood volume [36]. The compressed splanchnic reservoir transfers blood from the unstressed compartment to the stressed compartment, thereby increasing the mean systemic filling pressure through this mechanical mechanism, further amplified by sympathetic-adrenal activation. Additionally, hepatic compression due to diaphragmatic descent can distort the hepatic vascular geometry and increase resistance to portal blood flow, particularly under conditions of elevated lung volumes [28]. Furthermore, diaphragmatic descent and the resulting distortion of the geometry of the large veins (e.g., inferior vena cava) can increase the resistance to venous return, elevate the critical closing pressure, and reduce the maximum achievable venous return, likely due to hyperinflation of the right lung lobes [16]. Thus, an alternative mechanism of abdominal venous congestion is proposed, which could be amplified in clinical conditions of low lung recruitability and high PEEP levels, exacerbating abdominal venous stasis.

### Clinical evaluation of splanchnic congestion

It was proposed that abdominal venous congestion can be detected by evaluating venous Doppler signals using ultrasonography. These new findings have led to developing a scoring system known as the Venous Excess Ultrasound Score (VExUS), which quantifies the degree of congestion based on venous flow characteristics. This method has been validated in patients undergoing cardiac surgery and demonstrated a significant correlation with the development of AKI [37]. Moreover, in patients with chronic heart failure, abnormalities in the Renal Venous Stasis Index, assessed using Doppler ultrasound, have been identified as leading predictors of long-term adverse clinical outcomes [38]. However, the relationship between the VExUS score and the development of AKI has been underexplored in intensive care unit (ICU) patients, and the available data are controversial. In a study conducted on critically ill patients, the loss of continuous venous flow in the renal vein, which is indicative of renal congestion, was not associated with AKI or increased mortality at 28 days [39]. Conversely, the VExUS score was associated with AKI in a cohort of hospitalized ICU patients [40]. Therefore, given the controversial information surrounding this diagnostic method, it is currently not possible to definitively assert that renal venous Doppler abnormalities are associated with AKI in critically ill patients.

### Acute kidney injury associated with PEEP levels

In recent years, increased emphasis has been directed explicitly towards the deleterious effects of high PEEP levels on renal function. This connection has been firmly established in a broad range of preclinical and clinical studies.

The effects were investigated in pigs under mechanical ventilation, where higher PEEP levels were associated with increased AKI severity, observed at both 30 min and 48 h of evaluation [22]. Higher PEEP levels elevated the central venous pressure (CVP) and reduced the mean perfusion pressure (MPP), increasing fluid and sodium retention. This also significantly contributed to kidney and liver edema. Likewise, CVP was significantly higher in the groups with more severe AKI at 48 h, reaching values of 15 ± 5 mmHg in the RIFLE 3 group (renal failure), compared to 10 ± 4 mmHg in subjects without AKI. Concurrently, MPP decreased with the severity of AKI, reaching 46 ± 10 mmHg in the RIFLE 3 group versus 61 ± 12 mmHg in the group without AKI. Although it was possible to observe an association between changes in PEEP and the effects on CVP and MAP, direct causality with AKI could not be established. The relationship between the PEEP levels and renal fluid accumulation was evaluated using the wet/dry ratio, which measures the weight of a wet tissue sample relative to its fully desiccated weight after drying at 50 °C for 24 h. The lowest wet/dry ratio was observed at a PEEP level of 9 cmH₂O, whereas the highest ratio was recorded at 18 cmH₂O, indicating greater renal edema at higher PEEP levels. However, this trend was not linear, as similar wet/dry ratio values were observed at PEEP levels between 10 and 13 cmH_2_O. These effects help to better understand and detect potential pathophysiological mechanisms, and their findings suggest that venous congestion and reduced renal perfusion pressure could be relevant mechanisms in the development of AKI during MV [22].

Regarding clinical studies, the probability of developing AKI was secondarily examined in a meta-analysis that included seven clinical studies that compared the use of high versus low PEEP in critically ill patients [1]. The use of higher PEEP levels compared to low PEEP levels was not significantly associated with an increased risk of AKI. However, the variability in how and when AKI was measured in each study and the differences in the criteria for defining AKI introduced significant interpretation limitations. Additionally, the methods used to set PEEP were based on the PEEP/FIO_2_ table, which was not specifically designed to assess the effects of PEEP on lung recruitment. This heterogeneity in the definitions, timing of measurements, and form of PEEP settings makes it difficult to draw definitive conclusions about the impact of PEEP levels on the incidence of AKI [1].

Numerous observational studies have supported the relationship between lung function and the development of AKI, with the main proposed mechanism being again the relationship between high airway pressure and venous congestion (Table 1) [41–52]. In addition, we detail the physiological mechanisms that could be involved in developing AKI when using high values of PEEP.

**Table 1:** Association between PEEP setting and AKI development

Journal/Year	Population	Design	Event of interest	Results	Conclusions
Annals of Translational Medicine, 2019 (Tavares Leite et al.)	1.142 patients with ARDS	Retrospective cohort	Acute renal injury (AKI) was defined using serum creatinine levels and urinary output criteria according to the KDIGO criteria (Kidney Disease Improving Global Outcomes) stages I to III	Compliance of the respiratory system (Crs) and positive end-expiratory pressure (PEEP) were associated with severe AKI. Every 1 cmH_2_O increase in PEEP increased the risk of AKI by 5% (OR 1.05, 95% CI 1.03–1.10, p < 0.001). Median PEEP was 7.6 cmH_2_O, IQR 6.9–9.4	Crs reduction and PEEP increase were the only respiratory variables directly associated with severe AKI
American Journal of Respiratory and Critical Care Medicine, 2020 (Chaibi et al.)	211 patients with COVID-19-associated acute respiratory distress syndrome (C-ARDS)	Retrospective cohort	AKI was defined using KDIGO criteria stage 3 within the first seven days following ICU admission	Severe AKI (KDIGO 3) occurred in 26% of the patients. The 28-day mortality rate was 56% in patients with AKI and 24% in those without AKI (*p* < 0.001). Severe injury was associated with higher PEEP (OR 3.54, 95% CI 1.74–7.37 in multivariate analysis). The median PEEP was 13 ± 2 cmH_2_O in patients with severe AKI and 11 ± 2 cmH_2_O in patients with non-AKI (*p* = 0.02)	Severe AKI is common in patients with ARDS and is associated with a high mortality rate. Patients who used higher levels of PEEP had a greater chance of developing AKI than those who used lower levels of PEEP
Journal of Critical Care, 2020 (Beurton et al.)	73 patients with ARDS	Retrospective cohort	Group 1: 20 patients with an average PEEP of 14 cmH_2_O Group 2: 53 patients with an average PEEP of 10–12 cmH_2_O. Outcome: Association between PEEP levels and AKI (KDIGO stage 3) development and the need for renal replacement therapy	The group with higher PEEP showed an increase in severe AKI (KDIGO 3) compared to the group with lower PEEP (75% vs. 40%, *p* = 0.007) and required renal replacement therapy (RRT) within 14 days. (45% in the high PEEP group vs. 21% in the low PEEP group; *p* = 0.038)	Lower PEEP may be associated with a lower risk of renal damage and reduce the need for RRT, compared to a higher PEEP
European Journal of Anaesthesiology, 2021 (Valk, Christel et al.)	933 patients with ARDS	Multicenter observational cohort	Group 1: high PEEP (n = 259) Group 2: low PEEP (n = 674), according to the ARDSnet PEEP/FiO_2_ tables After matching, 234 patients remained in each group (n = 468) Outcomes: AKI and the need for renal replacement therapy (RRT) at 28 days. AKI was defined according to the KDIGO criteria	In the high-PEEP group, the median PEEP was 14.2 cmH_2_O (IQR 13.0 16.0). In the low PEEP group, the median PEEP was 12.0 cmH_2_O (IQR, 10.0 to 14.0). In the matched cohort, AKI occurred in 55.6% of the high PEEP group vs. 45.9% of the low PEEP group (OR 1.49, 95% CI 1.02–2.18, *p* = 0.039). In the matched cohort, the need for RRT was 25.6% in the high PEEP group vs. 17.9% in the low PEEP group (OR 1.58, CI 95% 1.01–2.46, *p* = 0.045)	In ARDS, due to COVID-19, the use of high PEEP and increasing PEEP levels increases the incidence of AKI and the need for renal replacement therapy
J Crit Care. 2021 (Geri G et al.)	Total: 27.248 ICU patients No mechanical ventilation: 20,682 patients (75.9%) mechanical ventilation: 6,566 patients (24.1%):	Retrospective cohort	Peep group distribution: peep < 5 cmH2O peep 5–8 cmH2O peep > 8 cmH_2_O Outcomes: Worsening Renal Function (WRF) Definition of WRF: Onset of AKI or Worsening by at least one KDIGO category compared to the previous day	Direct Association on Day 1: For each 1 cmH2O increment in PEEP: Relative Risk Ratio (RRR) = 1.36 [95% CI: 1.16, 1.6] Association on Day 2: For each 1 cmH2O increment in PEEP: RRR = 1.17 [95% CI: 0.88, 1.56] Pathophysiological Mechanisms: PEEP affected renal function through: Increased central venous pressure (CVP), Decreased mean perfusion pressure (MPP) Generation of renal venous congestion	Increasing PEEP levels were associated with: - Decreased perfusion pressure - Elevated central venous pressure - Higher probability of Worsening Renal Function
Journal of Clinical Monitoring and Computing, 2022 (Fogagnolo et al.)	30 patients with ARDS	Prospective observational	A group of 15 COVID-19 patients treated with high PEEP levels (median 14, interquartile range 12–14 cmH_2_O) was analyzed and compared with a second group of 15 patients with 'classic' ARDS treated with lower PEEP (average, 10 cmH_2_O). Outcomes: Resistance index of the renal artery and renal venous flow pattern via ultrasound 24 h after initiating mechanical ventilation. Secondary outcomes: occurrence of AKI-KDIGO at two days post-evaluation of renal blood flow	The renal artery resistance index (RARI) was higher in the high PEEP group than in the low PEEP group (0.71 vs 0.64, *p* = 0.04). There was a linear correlation between PEEP and RARI in the high PEEP group (r^2^ = 0.31, *p* = 0.03) but not in the low PEEP group. Continuous renal venous flow was observed in 71% of the high PEEP group and 33% of the low PEEP group. The high PEEP group had more AKI cases (8/15, 53%) than the low PEEP group (5/15, 33%)	Under protective ventilation, arterial and venous renal blood flow is more significantly compromised in patients with high PEEP than those with low PEEP. Patients with a higher PEEP developed AKI more frequently
Journal of Nephrology, 2022 ( Ottolina et al.)	101 patients with C-ARDS	Retrospective cohort	Patients were divided according to the average level of PEEP applied during the first seven days of ICU stay: Low PEEP group (n = 31) at 9.6 [8.0–10.5] cmH_2_O; Medium PEEP group (n = 32) at 12.0 [11.2–12.7] cmH_2_O; and High PEEP group (n = 32) at 14.7 [13.7–16.7] cmH_2_O. Outcome: AKI was defined using KDIGO criteria stage 2–3	AKI occurred in 16%, 38%, and 59% of the patients in the low, medium, and high PEEP groups, respectively (*p* = 0.002). AKI was detected two days later (median, range 1–4 days) in the multivariable analysis; high PEEP increased the risk of AKI fivefold compared to low PEEP (OR 4.96, 95% CI 1.1–21.9, *p* < 0.05)	High PEEP levels in patients with C-ARDS were associated with a five-fold increased risk of AKI and higher mortality
Critical Care Explorations, 2022 (Vemuri et al.)	4,484 patients required mechanical ventilation	Retrospective cohort	AKI during invasive mechanical ventilation	Plateau Pressure (cmH_2_O): Without AKI, 17 [14.5–19.3] cmH2O. With AKI: 19 [16.5–23.3] cmH_2_O. Compliance with the respiratory system: 47 [38.0–60.0] mL/cmH_2_O without AKI. With AKI: 40 [28.7–49.4] mL/cmH_2_O. Driving Pressure: Without AKI, 11.0 [IQR 9–13] cmH_2_O. With AKI, 13.0 [IQR 10–17] cmH_2_O. The study did not specify the level of PEEP that was used	Patients with AKI had a higher plateau and driving pressures, as well as lower compliance of the respiratory system, compared to those without AKI
Critical Care, 2023 (McNicholas et al.)	1.699 patients with C-ARDS	Multicenter observational cohort	AKI KDIGO stages 1–3 observed within the first 48 h of invasive ventilation	AKI within the first 48 h was less frequent in COVID-19 (21%) compared to non-COVID ARDS (54%). No association was found between high PEEP and AKI	No association was found between the PEEP level and AKI
Cureus, 2023 ( Yokoyama et al.)	151 patients with ARDS	Retrospective multicenter cohort	AKI was defined using SOFA score. Renal function was evaluated on days 1 and 4 post-admission	In multivariable analysis, the difference in PEEP between days 1 and 4 was positively associated with AKI (OR 1.123, 95% CI 1.017–1.240, *p* = 0.022). The median PEEP difference was 2 cmH2O in the group with AKI versus those without AKI. In the propensity analysis, higher PEEP values showed a trend towards a higher risk of AKI: PEEP difference > 5 cmH2O: OR 3.277 (95% CI 0.940–11.425), *p* = 0.065	PEEP can be a risk factor for worsening of renal function. However, no cutoff value for the PEEP difference was determined
Journal of Clinical Monitoring and Computing, 2024 (Fogagnolo et al.)	92 patients requiring mechanical ventilation for ≥ 48 h in the ICU	Prospective observational study	AKI occurrence and changes in renal resistive index (RRI) at different PEEP levels	AKI occurred in 30% (28/92) of patients. RRI increased from 0.62 ± 0.09 at PEEP 5 to 0.66 ± 0.09 at PEEP 15 (*p* < 0.001). Mean RRI during PEEP trial predicted AKI with AUROC = 0.834 [95% CI 0.742–0.927]. Patients with RRI ≥ 0.70 at least once during PEEP trial had 55% incidence of AKI vs 13% in others (*p* < 0.001)	RRI appears able to predict the risk of AKI in mechanically ventilated patients. RRI values are influenced by the PEEP level applied. Repeated RRI measurements at different PEEP levels may help assess the impact of PEEP on renal hemodynamics
Blood Purification, 2024 (Zacchetti et al.)	144 patients with C-ARDS	Retrospective cohort	AKI was defined according to KDIGO criteria, using serum creatinine and urine output. Patients who developed AKI at any stage within the first 15 days were included in the "AKI" group, while the rest were in the "no-AKI" group	In the univariate analysis, both PEEP and plateau pressure levels were significantly higher in the AKI group compared to the no-AKI group (*p* < 0.05). In the multivariate logistic regression analysis, only plateau pressure and not PEEP were independently associated with the development of AKI (OR 1.40, 95% CI 1.12–1.76, *p* = 0.003). PEEP ≥ 14 cmH_2_O was not associated with AKI (*p* = 0.8). A plateau pressure of ≥ 27 cmH_2_O increased the risk of AKI by 40% (OR 1.4, 95% CI 1.12–1.76, *p* = 0.003)	Plateau pressure, but not PEEP, was independently associated with AKI in COVID-19 patients

### Postulated pathophysiological mechanisms of PEEP-related renal congestion and AKI

#### Potential effects of PEEP on kidney elasticity

Because the renal parenchyma is enclosed within a rigid capsule, blood stagnation can reduce the elasticity of Bowman's capsule, reducing its compliance and increasing intrarenal pressure. This hypothesis is based on experimental studies in pigs investigating the hydraulic behavior of kidneys throughout a pressure–volume curve, introducing the concept of Intrinsic Renal Compartment Syndrome [53]. Next, in an ischemia–reperfusion model, the release of intrarenal pressure due to decompressive surgery improves renal tissue oxygen pressure, renal lactate release, regional delivery, consumption, and extraction of oxygen after reperfusion, resulting in a marked attenuation of structural damage and apoptosis, and improved renal function [54]. In other animal models of renal congestion due to renal vein occlusion, an increase in renal lymphatic drainage was observed as a compensatory mechanism to reduce renal pressure and edema [55, 56]. Likewise, it is interesting to mention that prior to the development of dialytic therapies (1901–1944), surgical decompressive capsulotomy was considered a treatment for severe renal failure, with survival of almost 70% in more than 2000 patients, which further highlights the relevance of renal congestion in the progression of AKI [57]. This clinical evidence highlights how the release of intrarenal pressure, and consequently, greater intrarenal compliance, can influence clinical outcomes.

#### Neurohumoral effects of PEEP

The application of PEEP can induce significant changes in the interface between the cardiovascular and renal systems through various compensatory neurohormonal mechanisms intended to preserve vital organ perfusion pressure [58]. One of the primary hemodynamic effects of PEEP is the reduction in cardiac output, which decreases renal blood flow sensed by the macula densa cells releasing renin and triggering the activation of the renin–angiotensin–aldosterone system, ultimately leading to the production of angiotensin II and III. This response begins when the macula densa senses a decrease in blood flow, releasing renin by juxtaglomerular cells and subsequent production of angiotensin II and III. These hormones increase sodium and water retention via aldosterone, reducing sodium excretion and increasing fluid retention, which exacerbates renal venous congestion [59]. Additionally, PEEP can elevate the levels of antidiuretic hormone (ADH) [59], which promotes water reabsorption in the renal collecting ducts contributing to further fluid retention. Experimental studies in animal models have shown that PEEP-induced ADH elevation occurs independently of lung volume, left ventricular transmural pressure, and serum osmolality, even under stable hemodynamic conditions and without arterial pressure changes. The exact mechanisms underlying this controversial effect remain incompletely elucidated. However, multiple physiological factors, including arterial baroreceptor activity, could influence ADH release in response to increased PEEP levels [60, 61].

However, as a compensatory response, atrial and cerebral natriuretic peptides are released to mitigate this effect by promoting natriuresis and inhibiting the renin–angiotensin–aldosterone system [59]. These neurohormonal mechanisms act together, and the predominance of these opposing neuroendocrine effects will depend on the magnitude and duration of the activating stimulus. It is crucial to recognize that the extent of these effects can vary based on the effect of PEEP, underscoring the importance of its careful and individualized adjustment in clinical practice to avoid hemodynamic compromise, fluid retention, and renal function impairment [58, 59].

#### Effects of PEEP on renal oxygen delivery and consumption

Kidney oxygen demand is comparatively lower than that of organs such as the heart or brain and is efficiently managed owing to its ability to extract oxygen [62]. Despite a considerable renal blood flow of approximately 1000 mL/min and an arterial blood oxygen content of 200 mL/L, the kidneys use only approximately 7% of the total oxygen consumed by the body, translating to an oxygen consumption of 7–8 mL/min. This level of consumption is crucial for the primary functions of filtration and reabsorption rather than intensive metabolic activities [63].

Renal oxygen extraction can increase secondary to a reduction in renal blood flow (with unchanged renal metabolism), an increase in renal metabolism, or both. When renal blood flow decreases without a change in renal metabolism, the kidney increases its oxygen extraction rate to compensate for the reduced supply. Oxygen extraction also increases if renal metabolism increases while the flow remains constant [62]. However, in cases of renal congestion, together with impaired renal blood flow, higher levels of oxygen delivery could be required owing to the increased diffusion distances caused by edema and tissue damage [63]. This vulnerability is explained by the countercurrent exchange of oxygen in the vasa recta, which limits oxygen delivery to the deep medulla, and the high metabolic and transport activity in the thick ascending limb of the loop of Henle, which increases the oxygen demand. This results in an oxygen supply–demand mismatch under conditions of renal hypoperfusion. Hypoxia in the thick ascending limb can lead to selective cellular damage and necrosis (as observed in experimental renal ischemia models), tubuloglomerular feedback activation, reduced glomerular filtration rate and potentially the onset of ischemic acute renal failure. To protect itself, the kidney can reduce cortical blood flow during hypotension to preserve medullary perfusion and decrease the glomerular filtration rate, thereby reducing the workload and oxygen demand of the thick ascending limb of Henle's loop. Furthermore, the countercurrent arrangement of the medullary blood vessels allows an efficient oxygen diffusion from the arteries to the veins. However, it results in an outer medulla with reduced oxygenation and deficient tissue PO_2_ levels (10–20 mmHg) compared with the renal cortex (50 mmHg) [64]. This condition places the renal medulla at a constant risk of hypoxia, making it vulnerable to damage during episodes of hypoperfusion or hypoxemia, such as it may occur during major surgery or shock states [65, 66].

### Final comments

**Fig. 3:**
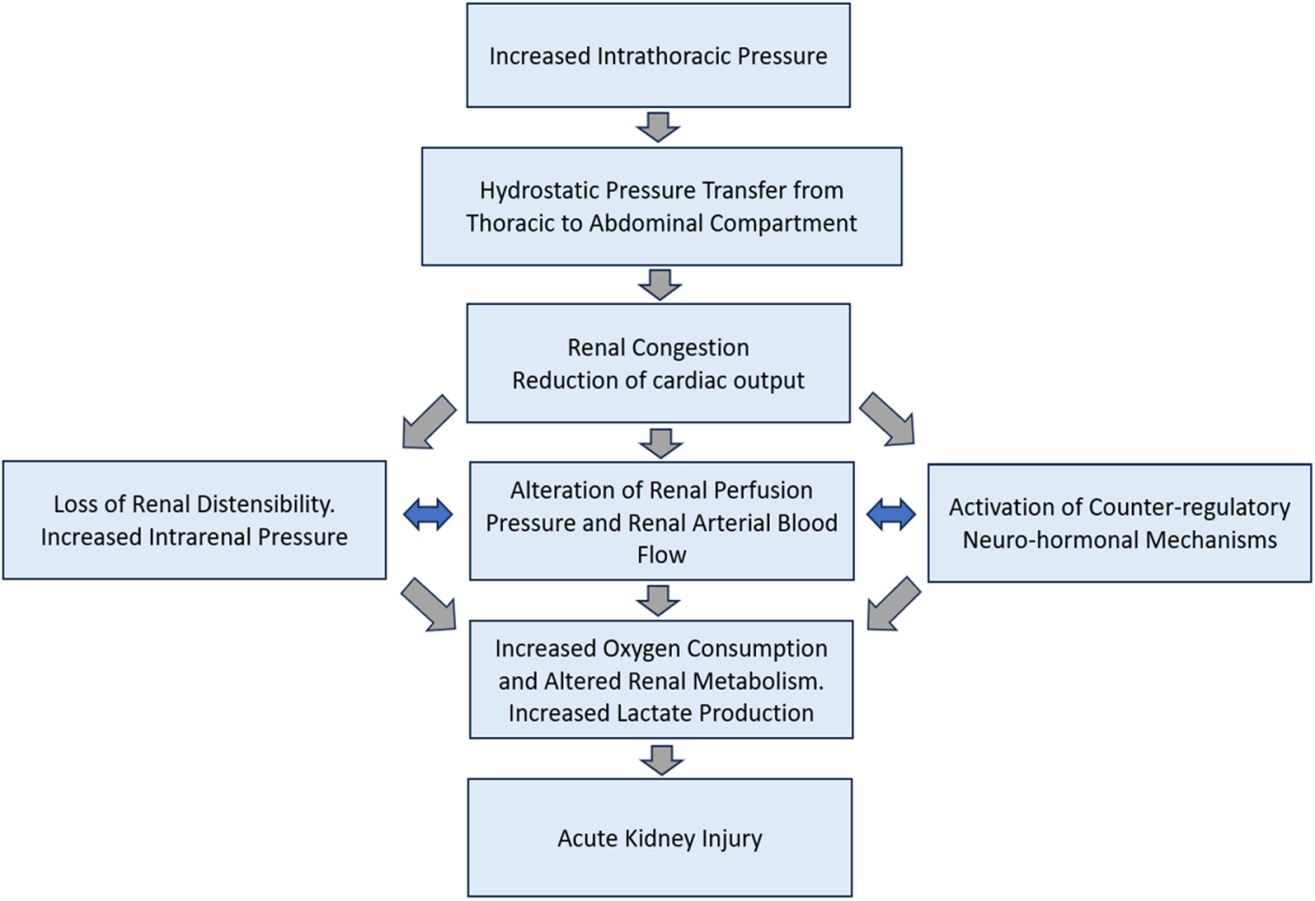
Proposal on how inadequately high PEEP levels affect renal perfusion, lead to venous congestion and impact renal function

The precise mechanisms linking lung and AKI remain unclear; however, three primary pathways have been proposed to explain lung-kidney crosstalk during MV: gas exchange abnormalities, distant effects of mechanobiology’s mechanisms triggered into the lungs (biotrauma), and hemodynamic changes, including impaired venous return and reduced cardiac output. Elevated PEEP increases the intrathoracic pressure, which can be transmitted to the abdominal compartment via the venous system. This process reduces the venous return and could favor abdominal congestion. Additionally, it can decrease cardiac output, reducing renal blood flow and perfusion pressure. In this way, a theoretical challenge arises in determining whether these effects are exclusively attributable to the use of high PEEP levels, regardless of respiratory system compliance, or whether they result from pulmonary overdistension, which can occur even at lower PEEP levels in lungs with a pressure–volume curve shifted towards the steep portion of the curve. Therefore, it has been proposed that the effects of PEEP on renal function can depend on both the absolute value of PEEP (high or low) and the pulmonary response to these levels (high or low recruitability). Thus, we propose to bridge this knowledge gap by focusing on the effects of PEEP on renal perfusion, renal venous congestion, and, ultimately, how these factors affect renal function (Fig. 3).

Elucidating the physiological mechanisms that explain why high PEEP levels in patients with ARDS can lead to AKI is crucial in clinical practice. This highlights the importance of always considering the cross-talk between organs and systems in critically ill patients. In this way, future studies should consider adjusting PEEP based not only on respiratory effects or cardiopulmonary interactions but also from a lung-heart-kidney perspective. In addition, the inherent variability of ARDS emphasizes the need to tailor mechanical ventilation strategies according to the specific characteristics of each patient. This personalized approach, grounded in the principles of precision medicine, is increasingly being recognized and embraced in clinical practice. Therefore, it is crucial not to overlook monitoring the splanchnic venous system when adjusting PEEP settings.

A limitation of this review is its reliance on a diverse range of sources, including experimental animal models, observational clinical studies, and interventional trials. Although this approach provides a comprehensive descriptive synthesis, it may restrict the ability to draw definitive conclusions. Nevertheless, integrating these findings highlights compelling opportunities for future research to deepen our understanding of personalized PEEP settings, taking into account not only pulmonary effects but also the physiological adverse effects that occur in distant organs.

## Conclusions

Inadequate PEEP settings in compliant lungs induce lung distension, which may contribute to renal congestion through vena cava compression. In addition, the retrograde transmission of pressure from the right atrium to the abdominal veins and impairment of renal blood flow are complementary mechanisms that may induce AKI. These findings underscore the need for rigorous research to validate these observations and to inform the development of strategies that optimize PEEP settings, balance respiratory benefits, and mitigate adverse renal effects.

## Data Availability

No datasets were generated or analysed during the current study.

## Abbreviations

MV: Mechanical ventilation
AKI: Acute kidney injury
PEEP: Positive end-expiratory pressure
ARDS: Acute respiratory distress syndrome
VILI: Ventilator-induced lung injury
R/I: Recruitment-to-inflation
MSFP: Mean systemic filling pressure
vr: Venous resistance
MAP: Mean arterial pressure
VexUS: Venous Excess Ultrasound Score
ICU: Intensive care unit
PIA: Intra-abdominal pressure
CVP: Central venous pressure
MPP: Mean perfusion pressure
ADH: Antidiuretic hormone
PaO_2_/FIO_2_: PaO_2_ over the fraction of inspired oxygen
C-ARDS: COVID-19-associated acute respiratory distress syndrome
C_RS_: Respiratory system compliance
